# Can the Bacterial Community of a High Arctic Glacier Surface Escape Viral Control?

**DOI:** 10.3389/fmicb.2016.00956

**Published:** 2016-06-21

**Authors:** Sara M. E. Rassner, Alexandre M. Anesio, Susan E. Girdwood, Katherina Hell, Jarishma K. Gokul, David E. Whitworth, Arwyn Edwards

**Affiliations:** ^1^Institute of Biological, Rural and Environmental Sciences, Aberystwyth UniversityAberystwyth, UK; ^2^Department of Geography and Earth Sciences, Aberystwyth UniversityAberystwyth, UK; ^3^School of Geographical Sciences, Bristol Glaciology Centre, University of BristolBristol, UK; ^4^Institute of Ecology, University of InnsbruckInnsbruck, Austria

**Keywords:** glacier, meltwater, virus, viral shunt, bacterial diversity, vesicles

## Abstract

Glacial ice surfaces represent a seasonally evolving three-dimensional photic zone which accumulates microbial biomass and potentiates positive feedbacks in ice melt. Since viruses are abundant in glacial systems and may exert controls on supraglacial bacterial production, we examined whether changes in resource availability would promote changes in the bacterial community and the dynamics between viruses and bacteria of meltwater from the photic zone of a Svalbard glacier. Our results indicated that, under ambient nutrient conditions, low estimated viral decay rates account for a strong viral control of bacterial productivity, incurring a potent viral shunt of a third of bacterial carbon in the supraglacial microbial loop. Moreover, it appears that virus particles are very stable in supraglacial meltwater, raising the prospect that viruses liberated in melt are viable downstream. However, manipulating resource availability as dissolved organic carbon, nitrogen, and phosphorous in experimental microcosms demonstrates that the photic zone bacterial communities can escape viral control. This is evidenced by a marked decline in virus-to-bacterium ratio (VBR) concomitant with increased bacterial productivity and number. Pyrosequencing shows a few bacterial taxa, principally *Janthinobacterium* sp., dominate both the source meltwater and microcosm communities. Combined, our results suggest that viruses maintain high VBR to promote contact with low-density hosts, by the manufacture of robust particles, but that this necessitates a trade-off which limits viral production. Consequently, dominant bacterial taxa appear to access resources to evade viral control. We propose that a delicate interplay of bacterial and viral strategies affects biogeochemical cycling upon glaciers and, ultimately, downstream ecosystems.

## Introduction

The surfaces of glaciers and ice sheets, which account for 11% of Earth’s land surface area, represent a recently discovered major freshwater ecosystem (Hodson et al., 2008; Irvine-Fynn and Edwards, 2014; Edwards et al., 2014a). During the melting season, conditions at the ice–atmosphere interface of the glacier surface are conducive to microbial activity since the flux of solar energy (Cook et al., 2010) and aeolian organic and inorganic matter (Stibal et al., 2008; Edwards et al., 2013b) to the ice supports phototrophic (Lutz et al., 2015) and heterotrophic metabolism (Hodson et al., 2007). Melting snowpacks, surficial algal biofilms and microbe–mineral aggregates (cryoconite) which darken the ice surface are now recognized (if ephemeral or patchy) foci of biodiversity and activity (Hell et al., 2013; Lutz et al., 2015).

Furthermore, the bare glacier ice surface itself comprises a seasonally dynamic three-dimensional microbial habitat recently defined as the glacial photic zone (Edwards et al., 2014a, Irvine-Fynn and Edwards, 2014). Penetration of solar radiation weathers the glacial ice crystals within the upper 1–2 m of the surficial ice crystal matrix, thereby forming a porous ice layer known as the weathering crust (Müller and Keeler, 1969), through which cells, particulates, and dissolved nutrients may percolate. Irvine-Fynn et al. (2012) presented flow cytometric data which demonstrated a net accumulation of cells within the weathering crust of a High Arctic glacier and that the supraglacial export of cells was impeded inversely with the rate of meltwater discharge under normal conditions. This presents a feedback mechanism for the accumulation of biomass and “biological darkening” (Irvine-Fynn et al., 2012) of the ice–atmosphere interface. Moreover, the glacial photic zone may harbor up to 1 × 10^21-26^ cells worldwide (Irvine-Fynn and Edwards, 2014), which is comparable to the oceanic photic zone, with an estimated 1 × 10^25^ cells (Whitman et al., 1998). Therefore, exploring the ecology of this poorly defined habitat is merited given its potential scale and roles in ice surface albedo, melting, and biogeochemical cycling (Edwards et al., 2014b).

It is known that viruses are abundant in supraglacial environments (Säwström et al., 2002) and that viral lysis of cells is an important cause of bacterial mortality in cryoconite ecosystems (Bellas et al., 2013). Cryoconite ecosystems exhibit high virus-like particle: bacterium ratios (*hereafter* VBR) and the abundances of virus-like particles (*hereafter* VLPs) and bacterial cells are closely correlated (Anesio et al., 2007). Unusually, high frequencies of visibly infected cells and very low burst sizes (BS, Säwström et al., 2007) are measured in Arctic cryoconite hole supernatant waters, indicating the importance of viral shunting of the microbial loop in supraglacial carbon cycling (Anesio et al., 2007; Säwström et al., 2007). Furthermore, evidence from reciprocal transplant experiments demonstrates that viruses from cryoconite can infect bacteria in downstream habitats (Bellas et al., 2013). However, less is known of the viral dynamics and interactions with the bacterial population of the glacial photic zone itself. Consequently, given the general significance of virus-induced bacterial mortality in supraglacial habitats and the importance of glacial-derived organic carbon for downstream terrestrial or coastal environments (Hood et al., 2009; Wilhelm et al., 2013), addressing the lacunae in our understanding of virus-mediated transfer of cellular organic carbon to meltwater-soluble dissolved organic carbon in the weathering crust is necessary.

In this study, the viral control of bacterial populations of supraglacial stream meltwater of a High Arctic glacier was investigated. Firstly, the rate and factors influencing viral decay in supraglacial meltwater were calculated. This permitted the estimation of the viral controls upon bacterial production in the glacial photic zone and the potential for the liberation of viruses from melting glacier surfaces to downstream environments. Secondly, we sought to understand whether changes in dissolved nutrient availability, for example, as a result of meltwaters of different provenance (Edwards et al., 2011, 2013a; Telling et al., 2011) or allocthonous nutrients, for example, those derived from proximal bird colonies (Zarsky et al., 2013) or atmospheric deposits (Hodson et al., 2010; Hell et al., 2013) dissolved in meltwater have an impact on virus–bacterium interactions and consequently bacterial abundance, productivity, and diversity within the weathering crust.

## Materials and Methods

### Site Description

Water samples were obtained from a moraine meltwater lake and a supraglacial meltwater stream associated with Midtre Lovénbreen (ML; 78.53°N, 12.05°E), a small but well-described (Säwström et al., 2002; Anesio et al., 2007; Cook et al., 2010; Edwards et al., 2011, 2013a, 2014b) Svalbard valley glacier (Map: **Supplementary Figure S1**). The moraine meltwater lake was situated in the fore field of ML, halfway between the snout of the glacier and the shore of Kongsfjorden Bay (78.9033°N, 12.0708°E). The lake (180 m long and 55 m wide) was ca. 5 m above a neighboring meltwater river, surrounded by moraine heaps, and lacked any visible macrophyte vegetation. The supraglacial meltwater stream was situated just below the snowline between stakes 3 (78.89034°N, 12.05450°E) and 4 (78.88660°N, 12.04864°E) of the Norwegian Polar Institute’s ablation stakes. The stream was ca. 40 cm wide at the time of sampling.

### Viral Decay Experiment

To estimate virus production in different glacial settings, a reciprocal transplant experiment was set up to compare the effects of temperature on the viral decay rates in water from supraglacial stream (*Stream*) and proglacial lake (*Lake*) habitats. Water from the two sources was incubated in quadruplicate at two locations: the moraine lake (*Warm*; temperature range: +9.9 to +10.9°C at times of sampling) and a supraglacial stream (*Cold*; temperature range: +0.4°C at times of sampling); giving a total of 16 experimental units (**Supplementary Figure S1**).

Water was collected on July 21, 2006, using plastic carboys washed with bleach, rinsed with Milli-Q water, and then pre-rinsed with approximately 5 L of sample water. Water was filtered through 0.2 μm filters (VacuCap 90 Filter Unit with Supor Membrane, PALL Life Sciences) to remove all organisms except for particles in the typical size range of viruses within 4 h of collection. Afterwards, 100 ml of water was transferred to UV-B-transparent, sterile polyethylene bags (Whirl-pak, Fisherbrand). Fresh viral production was prevented by filtration to exclude potential hosts and microcosms were monitored for contamination using SYBR epifluorescence microscopy. Triplicate samples of both filtered and unfiltered water were also taken for the estimation of bacterial and viral abundances in the two habitats. These samples were fixed immediately with 0.02 μm filtered 25% glutaraldehyde (final concentration approximately 1% v/v) and frozen within 4 h. Filtration, bag preparation, and return to the field were completed within 48 h, with bags being kept in the dark at +3°C in the meantime. Incubation *in situ* started on July 23, 2006 (Day 0). Samples were taken on Days 2, 5, 8, and 12 for enumeration of VLP and bacterial cells.

To investigate the decay dynamics of viruses in the two habitats, the model of Fischer et al. (2004) was applied using assumptions regarding BS (BS = 3; Säwström et al., 2007), carbon content of supraglacial bacteria (11 fg C cell^-1^; Priscu and Christner, 2004; Anesio et al., 2010), and viruses (0.2 fg C VLP^-1^; Suttle, 2005). Details of modeling are provided in full as Supplementary information.

### Nutrient Enrichment Experiment

To investigate viral and bacterial responses to nutrient fluctuation, supraglacial meltwater was amended with nutrients and the abundance of VLP, bacteria, and bacterial carbon production (BCP) were monitored over 12 days. Meltwater collected exactly as above on 21 July 2006 was filtered through GF/F (Fisherbrand) filters to exclude grazers, phytoplankton, and larger organisms. Six increments of nutrient concentration representing low, median, and high concentrations of C, N, and P measured in ML supraglacial meltwater and 2×, 4×, and 8× the highest concentrations measured by Säwström et al. (2002) were added as 0.02 μm filtered stocks as detailed in **Table 1**. Four (450 mL-volume) replicates of each treatment and non-amended controls were incubated in the dark at +3°C and subsampled aseptically on Days 2, 4, 7, 10, and 12 for measurement of VLP and bacterial abundance (2–5 mL), and for BCP (1.7 mL) on days 2, 4, 7, and 10. Samples (40 mL) for scanning electron microscopy (SEM) were collected and fixed with VLP-free glutaraldehyde on days 7 and 10.

**Table 1:** Enrichment scenarios for nutrient amendment experiment.

	NA CtrI	I	II	III	IV	V	VI
PO_4_-P (μg l^-1^)	–	1	5	10	20	40	80
NH_4_NO_3_-N (μg l^-1^)	–	6	11	22	45	90	180
glucose- C (mg l^-1^)	–	0.125	0.25	0.5	1	2	4
Addition equal to^∗^	(no addition)	lowest	mid-range	highest	2 × highest	4 × highest	8 × highest


*^∗^Values for ML meltwater reported by Säwström et al. (2002).*

### Bacterial and Viral Enumeration

For enumeration of bacteria and VLP, samples were analyzed by epifluorescence microscopy, a method used routinely to provide measurements of viral abundance in aquatic samples. Epifluorescence microscopy demonstrates both superior precision and robust correlation with other metrics of viral abundance, for example, transmission electron microscopy (e.g. Hara et al., 1991; Ferris et al., 2002) and its common usage supports comparison between habitats (Wommack and Colwell, 2000; Pearce and Wilson, 2003). Samples were filtered on the day of collection onto 0.02 μm pore size Anodiscs (25 mm, Whatman Anodisc 25) using a gentle vacuum and stained with SYBRGold (1:400 solution; Molecular Probes, Invitrogen Inc.) for 15 min in the dark (Noble and Fuhrman, 1998; Patel et al., 2007). Slides were treated with Antifade (SlowFade antifading kit, Invitrogen Inc.) and stored wrapped in Al-foil at –20°C until enumeration using an epifluorescence microscope (Zeiss Axioplan) at ×1000 magnification. When possible, 20 fields and a minimum of 100 bacterial cells and 100 VLPs per field were counted by one experienced operator, operationally defining VLPs as high intensity fluorescence “pinpricks” as per the criteria of Patel et al. (2007), prior to conversion into number of bacterial cells and VLPs mL^-1^ in the original sample. SEM was conducted on uranyl-acetate stained discs and imaged using a Hitachi S4700 II FE-SEM. Further details are provided as Supplementary information.

### BCP Estimation

Bacterial production was measured using the ^3^H-leucine incorporation method (Kirchman et al., 1985; Anesio et al., 2010). Briefly, L-[4,5-^3^H]-leucine (specific activity 166 Ci mmol^-1^; Amersham) was diluted with non-radioactive leucine and added to 2 ml screw–cap tubes containing 1.7 ml of supraglacial meltwater to a final concentration of 100 nM. Logistical restrictions prevented the use of trichloroacetic acid; therefore, 72 μl of glutaraldehyde (final concentration: 1% v/v) was used to stop all bacterial activity in killed controls by addition 30 min prior to ^3^H-leucine and in live samples after incubation on ice in a fridge at +3°C for 3 h after the addition of ^3^H-leucine. Activity in each sample was measured using a liquid scintillation analyzer (Tri-Carb 2500TR, Packard) and leucine incorporation rates converted to BCP as per Smith and Azam (1992).

### Bacterial Community Composition

At the end of the experiment, the microcosm bacterial communities were pooled by treatment and harvested on 0.2 μm filters, as was an initial sample of the source meltwater processed upon collection, hereafter termed the “descriptive” sample. Bacterial biomass trapped on filters was frozen immediately before return to the Aberystwyth laboratory frozen in insulated containers. Replicates were pooled by treatment to ensure that sufficient material (each pool comprising four replicates totaling ca. 1 l per treatment) could be concentrated onto the filters for DNA extraction. All steps before polymerase chain reaction (PCR) were conducted using aerosol resistant filter tips and certified DNA-free plasticware in laminar flow hoods.

In brief, filters were disrupted by bead-beating in a Biospec Mini-8 bead beater for 2 × 30 s as part of a PowerSoil DNA extraction kit (MoBio, Inc) procedure which was otherwise exactly as specified by the manufacturer. 454 amplicon pyrosequencing was performed on samples. Since the amplification of 16S rRNA gene fragments using barcoded primers proved unreliable (data not shown), a hemi-nested PCR strategy was employed using 15 cycles of PCR with bacterial 16S rRNA primers 27F (5′-AGAGTTTGATCMTGGCTCAG-3′) and 1389R (5′- ACGGGCGGTGTGTACAAG-3′). Subsequently, PCR products were barcoded using 27F (Roche B-tagged) and 357R (5′-CTGCTGCCTYCCGTA-3′; 5′-tagged with the Roche A and MID barcode tags). A microliter of PCR product amplified in 25 μL reactions comprising 10× reaction buffer, 1.8 mM MgCl_2_, 0.2 mM dNTPs, 0.2 μM of each primer, 1.25 U FastStart High Fidelity Enzyme Blend (Roche Biosystems), and subjected to 30 cycles of 30 s at 95°C, 30 s at 55°C, 2 min at 72°C and a terminal elongation of 7 min at 72°C. Negative controls were run alongside samples, and no amplification was observed. Amplicons were cleaned with Agencourt AMPure XP beads (Beckman-Coulter Genomics) before pyrosequencing using Titanium chemistry on the Aberystwyth University Roche GS-FLX 454 sequencer (Roche Diagnostics Ltd).

Amplicon pyrosequence data were analyzed using QIIME version 1.9 (Caporaso et al., 2010). Sequencer output was demultiplexed and a single fasta file with sample labels was created. The sequences were filtered by length and all sequences shorter than 120 nucleotides long were removed from the fasta file. Operational Taxomomic Units (OTUs) were picked using UCLUST. Representative OTUs were picked using the most abundant method and taxonomy was assigned based on 97% similarity using the Ribosomal Database Project (RDP) method and the GreenGenes database (gg_13_8_otus). Sequences were aligned using Pynast, after which chimeric sequences were identified using ChimeraSlayer and those discarded. All positions which had gaps in every sequence were removed. The resulting fasta file was used to make an OTU table. A filter was applied to remove any OTUs present at less than 0.01%. Finally, a single rarefaction to the lowest common number of reads per sample (here: 2930) was performed. To establish the extent of fine-grained variation in the composition of 97% identity OTUs, the process above was repeated as described but with 99% identity OTUs and rarefaction to the lowest common number of reads per sample (2970). A maximum likelihood phylogeny (Tamura et al., 2013) of selected 99% identity OTUs was constructed based upon MUSCLE-alignment (Edgar, 2004) of 144 sites along with the closest named relatives (CNR) of those OTUs identified by Basic Local Alignment Search Tool (BLAST). Pyrosequencing data are available from the European Bioinformatics Institute Short Read Archive (EBI-SRA, PRJEB9769).

## Results

### Viral Decay Rate

The rates of viral decay were compared in *Lake* and *Stream* waters incubated in quadruplicate as a reciprocal transplant experiment conducted over 12 days. Each experimental unit was monitored for bacterial growth (and thus for the potential replenishment of VLPs) after the initial filtration to exclude bacteria. One of the *Lake–Warm* experimental replicate with visible changes in cell number upon epifluorescence microscopy was excluded from further analysis, leaving three replicates for the *Lake–Warm* treatment.

**FIGURE 1:**
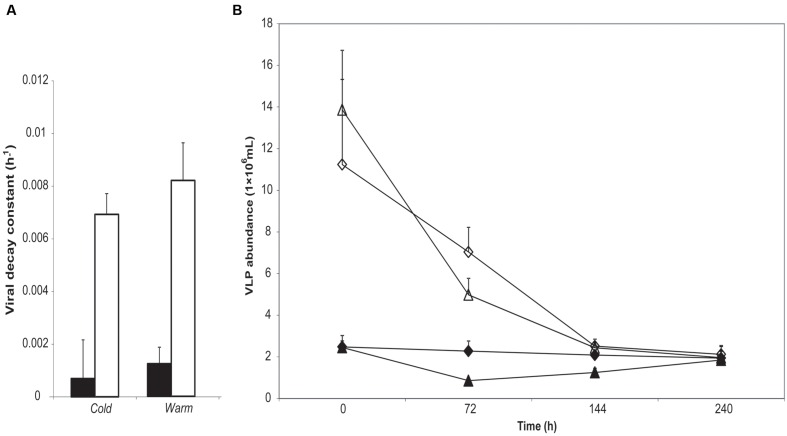
**(A)** Mean viral decay rate *k* (h^-1^) calculated from changes in virus-like particle (VLP) abundance over the course of 10 days incubation of either supraglacial stream water (gray bars) or proglacial lake water (white bars). **(B)** Changes in VLP abundance over 10 days of supraglacial stream water (dark symbols) and proglacial lake water (clear symbols) incubated in stream (*Cold*; diamonds) or lake (*Warm*; triangles). No effect of incubation temperature manipulation (by reciprocal transplant) is apparent, while the water source results in highly significant differences in *k* (Kruskal–Wallis: *H* = 22.93, *p* < 0.001). Error bars represent ±1 SD.

Application of viral abundance data to the exponential decay function (Fischer et al., 2004) permitted estimation of the viral decay constant *k* (given in the standard units of h^-1^ on the assumption of minimal diurnal variation in decay rate) for each replicate, as shown in **Figure 1**. Much lower rates of viral decay are apparent in supraglacial meltwater than proglacial lake water. Highly significant differences in *k* were returned by meltwater source (Kruskal–Wallis, *H* = 22.3; *p* < 0.001) but not incubation site (Kruskal–Wallis, *H* = 2.88; *p* = 0.090).

### Viral Control of Bacterial Production in Supraglacial Meltwater

The mean value of *k* for UV-exposed supraglacial meltwater incubated on the glacier surface (mean *k* = 0.0074 ± 0.0015 h^-1^) was therefore applied to model viral dynamics (Fischer et al., 2004) as detailed in Supplementary Methods. Bacterial secondary production estimated from independent incubations of glacial meltwater in triplicate conducted on 24 July 2006 yielded mean values of 17 ng C l^-1^ h^-1^ (±3 ng C l^-1^ h^-1^), within the range of previously reported values (Anesio et al., 2010). By using the product of mean pre-filtration, bacterial abundance of 0.05 × 10^6^ cells ml^-1^ and a standard conversion factor of 11 fg C cell^-1^ (Norland et al., 1987), bacterial biomass was estimated at 550 ng C l^-1^. Hence, mean meltwater bacterial growth rate (*μ*_b_) is 0.03 h^-1^, with an estimated community doubling time of 22 h. The rate of viral proliferation, *f*_v_ is estimated at 0.011 h^-1^ and consequently the viral control equals to 35.5% of bacterial secondary production within the supraglacial meltwater, some 6.1 ng C l^-1^ h^-1^.

### Nutrient Amendment Experiment Effects on Bacterial and VLP Abundance

**FIGURE 2:**
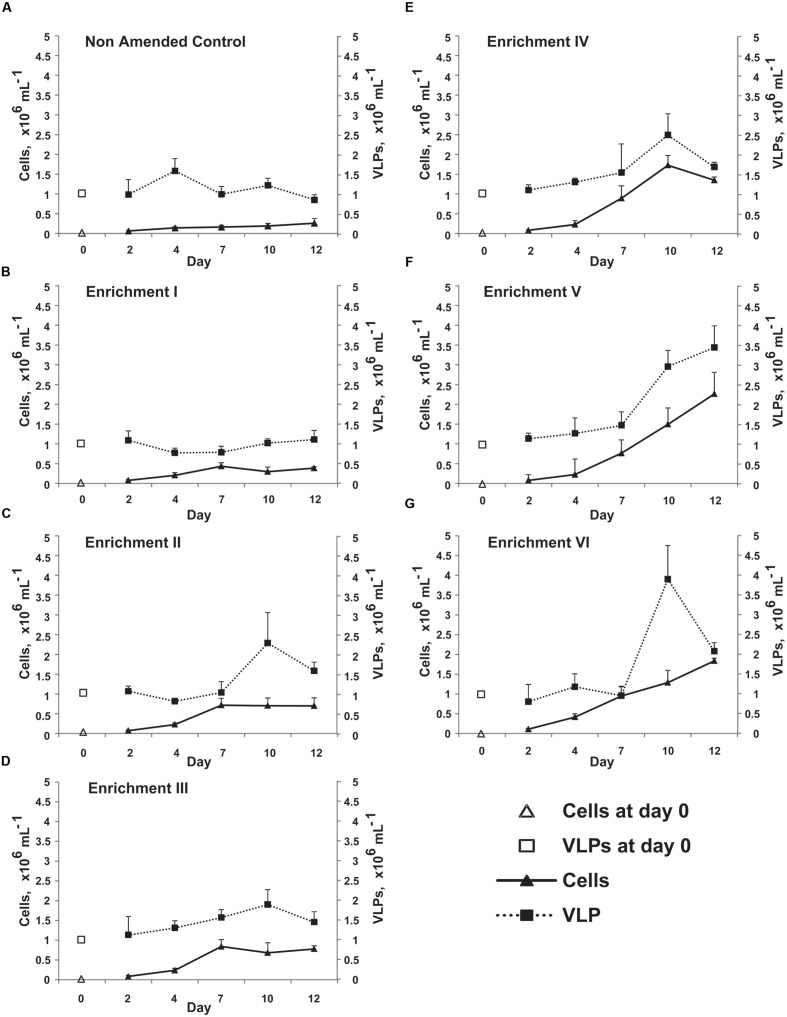
**Mean abundance of bacterial cells and VLPs enumerated by SYBR-gold staining and epifluorescence microscopy in the Non-Amended Control and Enrichments I–VI **(A–G)** from the nutrient amendment experiment.** Day-0 values represent the *in situ* bacterial and VLP abundance. Error bars represent ±1 SD.

Given the considerable pressure from viruses upon bacteria revealed above, we sought to understand how virus–bacterium interactions within the glacial photic zone respond to changes in nutrient availability. The nutrient amendment experiment revealed changes in bacterial and VLP abundance over 12 days’ incubation *ex situ* in the dark at +3°C (**Figure 2**). Bacterial abundance was significantly different between time points (repeated measures ANOVA; *f* = 396.67, *p* < 0.001) with a significant interaction between time and enrichment treatment (*f* = 8.48, *p* < 0.001), indicating an increase in bacterial abundance over time increasing with nutrient enrichment levels. *Post hoc* testing (Tukey’s Honest Significant Difference) of the enrichment factor revealed four non-overlapping homogenous subsets (*p* ≤ 0.05). The Non-Amended Control and Enrichment I had significantly lower bacterial abundance than the other treatments and were significantly different to each other. Enrichments II and III were significantly different to all other treatments (but not each other, *p* = 0.974), while Enrichments IV–VI were significantly different from other enrichments but not each other (*p* = 0.205). Viral abundance increased for all treatments over time (*f* = 48.02, *p* < 0.001) and between enrichment steps (*f* = 12.66, *p* < 0.001) with a highly significant interaction (*f* = 6.78, *p* < 0.001).

**FIGURE 3:**
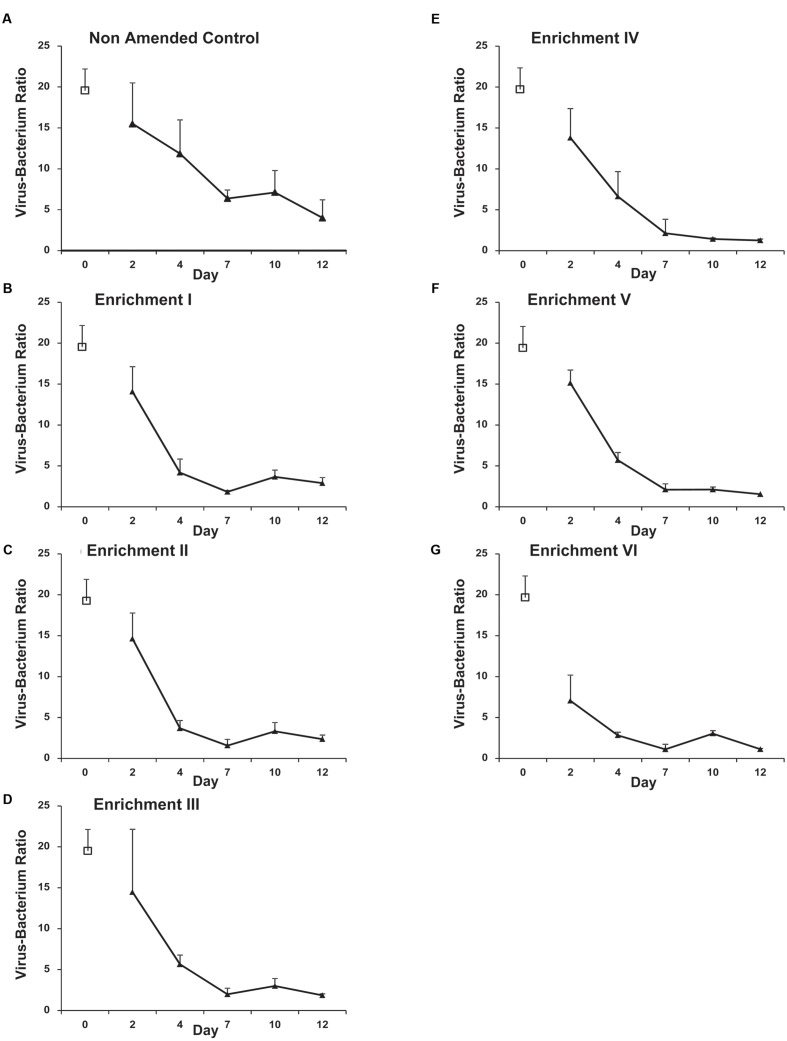
**Mean Virus-bacterium ratio (VBR) assessed by SYBR-gold staining and epifluorescence microscopy in Non-Amended Control and Enrichments I–VI **(A–G)** of the nutrient amendment experiment.** Day-0 values represent the *in situ* bacterial and VLP abundance. Error bars represent ±1 SD.

Nevertheless, a marked decline in the VBR from ca. 15 on Day 2 to between 1 and 4 on Day 12 was observed for all treatments (**Figure 3**). Correspondingly, a highly significant effect of time and enrichment step is observed (*f* = 41.07 and *f* = 30.72, respectively, both *p* < 0.001) with a highly significant interaction (*f* = 3.53, *p* < 0.001) demonstrating changes in VBR over time differed between enrichment treatments. Bonferroni pairwise comparisons revealed all time points differed significantly (*p* ≤ 0.001) except Days 7 and 12. The Non-Amended Control and Enrichment VI were highly significantly different from each other and all other treatments.

### Nutrient Amendment Effects on BCP

**FIGURE 4:**
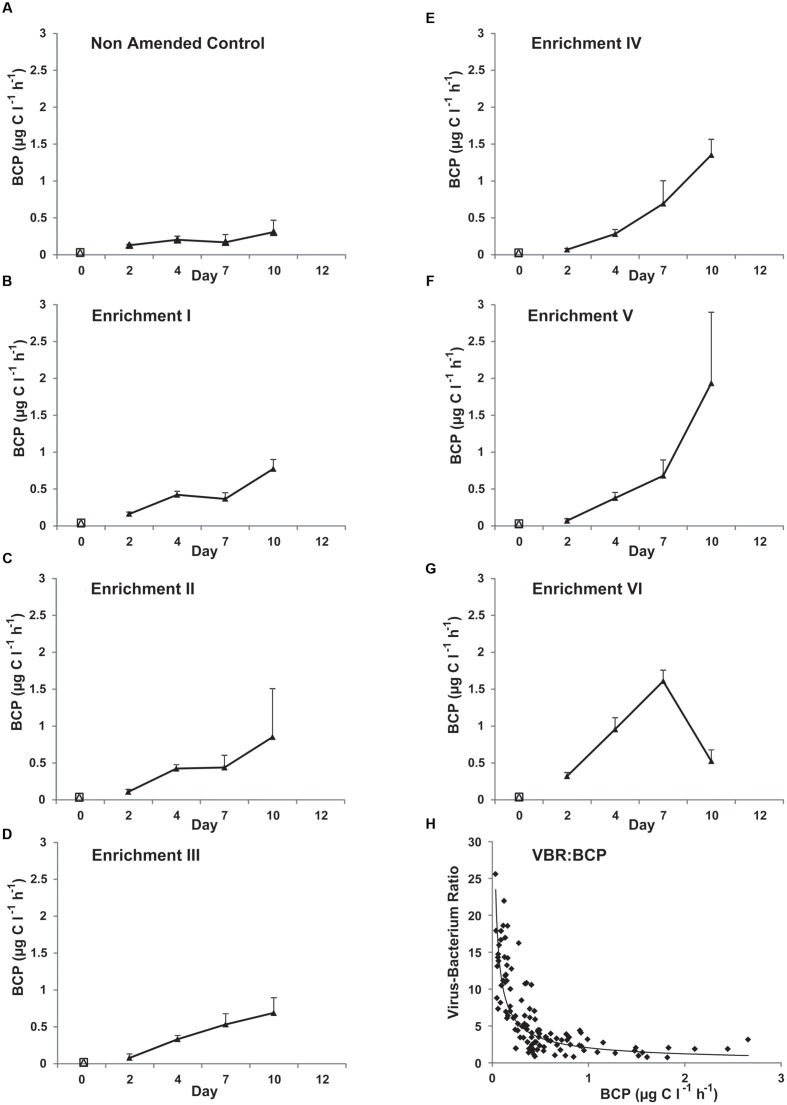
**Mean bacterial carbon production (BCP) as measured using ^3^H-leucine incorporation in Non-Amended Control and Enrichments I–VI **(A–G)** of the nutrient amendment experiment.** Day-0 values represent the *in situ* BCP measurement. Note that BCP could not be measured at Day 12 because of the limited availability of ^3^H-leucine. **(H)** The relationship between VBR and BCP across Days 2–10 for all treatments.

All treatments (**Figure 4**) demonstrated an increase in BCP over time (*f* = 30.81, *p* < 0.001; Bonferroni tests show all time points significantly different) and between enrichment steps (*f* = 25.53, *p* < 0.001). Values at the start of the experiment ranged between 0.03 and 0.07 μg C l^-1^ h^-1^ and between 0.31 and 1.94 μg C l^-1^ h^-1^ at Day 10. A significant interaction between both time and enrichment factors (*f* = 7.48, *p* < 0.001) was revealed. Pairwise comparisons revealed Non-Amended Control and Enrichment VI were significantly different from each other and all other steps (Bonferroni; *p* ≤ 0.004). Of note is the strong relationship apparent between declining VBR and increasing BCP between Days 2 and 10 (**Figure 4H**; Spearman rho = -0.81, *p* < 0.001) but not as apparent between VBR and mean bacterial growth rate (data not shown; Spearman rho = 0.29, *p* = 0.001).

### Effects on Bacterial Community Composition

**FIGURE 5:**
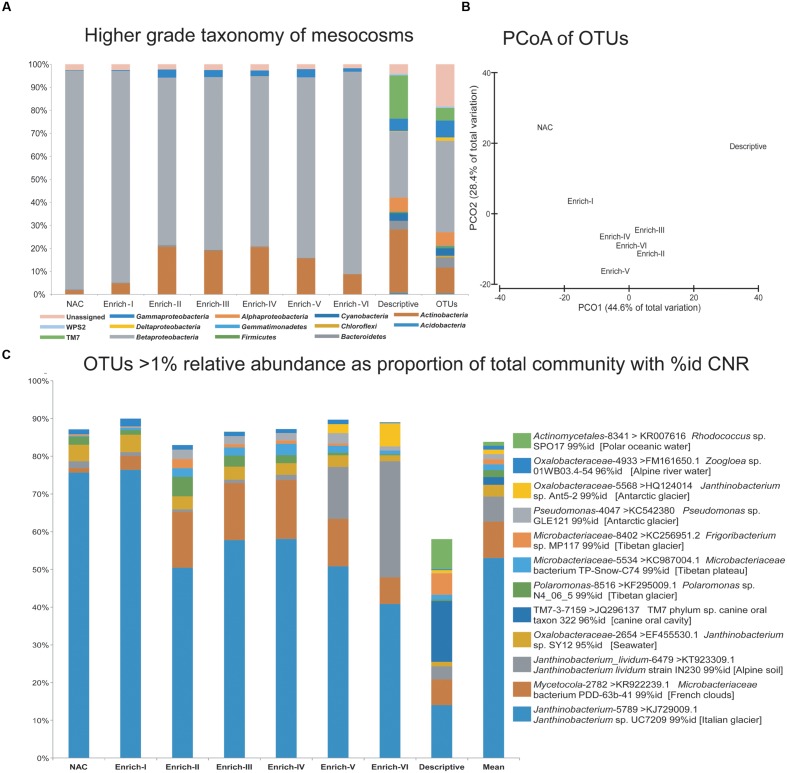
**Bacterial community composition at the termination of the nutrient enrichment experiment revealed by 16S rRNA gene amplicon pyrosequencing.** **(A)** The percentage of the community relative abundance assigned to higher grade taxa (phyla or proteobacterial class) in each of the microcosms and the descriptive sample collected at the start of the community. The “OTUs” column represents the number of OTUs assigned to each taxon. **(B)** Principal Coordinates Analysis (PCoA) of Bray Curtis similarities from fourth-root transformed OTU relative abundances. **(C)** The taxonomic assignment of all OTUs present at a mean relative abundance greater than 1% within the data set (“Mean” column). GreenGenes assignments at a confidence of 0.80 to the lowest grade taxon are provided with the *de novo* OTU ID (e.g. *Janthinobacterium_lividium*-6749), while the CNR of each OTUs representative sequence obtained via Basic Local Alignment Search Tool (BLAST) searching of non-environmental sequences is provided as NCBI accession number, taxon name and percent identity match.

The interactive effects of nutrient amendment and viral pressure on the bacterial community’s composition at the termination of the experiment were examined using pyrosequencing of the V1–V3 region of the 16S rRNA gene V1–V3 with each microcosm pooled according to treatment. Dilute templates necessitated hemi-nested PCR amplification, as previously experienced with supraglacial samples (Hell et al., 2013), which also has the additional benefit of minimizing barcode primer bias (Berry et al., 2011). Following processing, and rarefaction to 2,930 reads per sample clustered in 265 ≥ 97% id OTUs were assigned to the GreenGenes taxonomy. Across the data set, *Proteobacteria*, and specifically the class *Betaproteobacteria*, were consistently highly dominant, followed by the phylum *Actinobacteria* and eight other phyla (**Figure 5A**). Shifts in the community at the OTU level are apparent (principal coordinates analysis, PCoA; **Figure 5B**), with nutrient amended treatments clustering discretely from the pooled non-amended control and the descriptive sample; however, PERMANOVA of fourth-root transformed Bray–Curtis distances between samples tested using a model of non-amended (Descriptive and Non-Amended Controls) vs amendments within the range of nutrients measured in ML meltwater (Enrich I–III) vs amendments above the range of nutrients measured in ML meltwater is not significant (Pseudo-*F* = 1.4917, *p*(perm) = 0.0588).

Twelve OTUs are present at a mean relative abundance greater than 1% (**Figure 5C**), and account for 86.5–89.9% of the reads assigned to taxonomy in each of the Enrichment communities and 58% of the reads assigned to taxonomy in the *in situ* descriptive sample community. Across all pooled samples, Descriptive, Non-Amended Control and Enrichment, the communities are consistently dominated by the OTU *Janthinobacterium*-5789 (where 5789 represents the arbitrarily assigned *de novo* OTU reference for that OTU) which is assigned to *Janthinobacterium* at a confidence threshold of 0.8 by GreenGenes and accounts for 40.8–76.3% of all reads assigned to taxonomy. As the most dominant OTU in the more even descriptive community sample, *Janthinobacterium*-5789 accounts for 10.3% of the reads assigned to taxonomy. Of the 12 prominent OTUs, 10 possess CNR at 99% identity which were cultured from cryospheric samples, with the exception of *Oxalobacteraceae*-5568, which shows 96% identity to a *Zoogloea* sp. isolate from an Alpine river. Of the remaining two isolates, one shows 95% identity to a marine *Janthinobacterium* sp. isolate, while an OTU assigned to TM7 which is present in the descriptive community only shows 96% identity to a host associated taxon.

**FIGURE 6:**
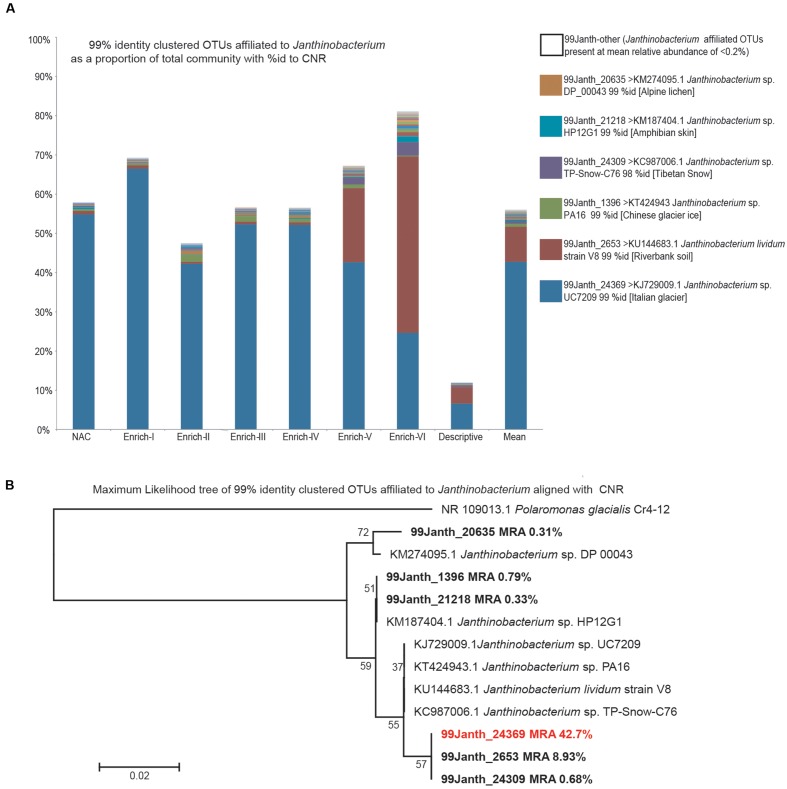
***Janthinobacterium* affiliated OTUs clustered at 99% identity.** **(A)** The percentage of the community relative abundance assigned to *Janthinobacterium* OTUs processed as above but with OTU clustering at 99% identity. The six OTUs present at a mean relative abundance >0.2% are named along with their CNR of each OTUs representative sequence obtained via BLAST searching of non-environmental sequences is provided as NCBI accession number, taxon name, and percent identity match. Thirty-one *Janthinobacterium* assigned OTUs individually present at a mean relative abundance <0.2% are shown individually but named as 99*Janth*-other. **(B)** Maximum likelihood phylogenetic tree of the six most abundant *Janthinobacterium* OTUs and their CNR, with the glacial *Betaproteobacteria* member *Polaromonas* as an out group over 144 positions aligned using MUSCLE.

Considering the prominence of *Janthinobacterium*, OTUs within the data set the potential for fine-grained variation (i.e. variation within 97% OTUs) as a marker for intraspecific dynamics within the *Janthinobacterium* genus was evaluated by re-processing the data set at the 99% identity OTU level. The bacterial community composition revealed by the re-analysis was highly congruent for the 97% identity OTU analysis. For 99% identity OTUs assigned to *Janthinobacterium* as above (**Figure 6**) the salient trends of 97% identity OTUs are recapitulated (**Figure 5**). Notably, a single 99% identity OTU assigned to *Janthinobacterium* (99*Janth*-24369) dominates the data set. Its representative sequence exhibits 100% identity to the 97% identity OTU Janthinobacterium-5769 and showing 99% identity to the same cryospheric isolate as *Janthinobacterium-*5789. Within Enrich-V and Enrich-VI pooled samples, a second *Janthinobacterium* affiliated 99% identity OTU (99*Janth-*2653) exhibits an increased relative abundance. Both 99*Janth-*24369 and 99*Janth-*2653 sit within the same clade of a maximum likelihood phylogeny (**Figure 6B**) of 99% identity *Janthinobacterium* OTUs present at a mean relative abundance >1% within the data set and their CNR.

**FIGURE 7:**
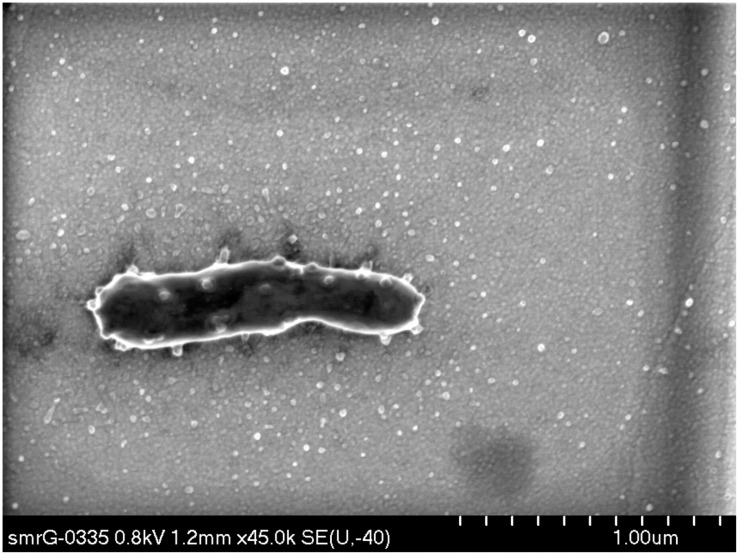
**Exemplar image of bacterium in nutrient-amendment experiment producing OMVs.** The FE-SEM micrograph is from Enrichment IV on Day 7 and is stained by 5% uranyl acetate for 3 min.

Coupled with the decline in VBR and the inverse relationship between VBR and BCP, the stable dominance of prominent OTUs suggests that viruses are unable to maintain their control of the bacterial population by the experiment’s conclusion. On the contrary, it appears that dominant taxa that can use the resources are able to retain their dominance of the community across a spectrum of nutrient enrichment scenarios. While only some samples from the experiment were available for SEM analysis, **Figure 7** illustrates a representative SEM micrograph of an individual bacterium in Enrichment III on Day 7 with numerous protrusions budding from the cell’s surface. This may represent one putative mechanism by which the bacteria may evade viral pressure in these circumstances, which is consistent with the appearance of outer membrane vesicles (OMVs), known to protect bacteria from viral attack (Biller et al., 2014). Although **Figure 7** is the most remarkable example, other SEM images reveal similar OMV features on bacterial cell surfaces within the experiment lending qualitative support to this notion; these are presented as **Supplementary Figure S2**.

## Discussion

This study sought to elucidate the viral control on bacterial communities within the photic zone of a High Arctic glacier and whether manipulation of resource availability could allow the bacterial community to escape that viral control. It should be noted that the findings in this study are principally constrained to the High Arctic glacier in question rather than a diverse range of glacial systems. Nevertheless, the glacier itself is very well described in terms of its ecology (Säwström et al., 2002; Anesio et al., 2007; Irvine-Fynn and Hodson, 2010) and could be considered a potential model site. It appears that VLPs in supraglacial meltwater are robust to decay, and that the consequent maintenance of high VBR in natural conditions prompts a mechanism for the control of over a third of BCP *in situ*, and that the bacterial community can use resources to evade viral pressure. The results are consistent with the dominance of defensive specialist taxa, presenting a paradox (Winter et al., 2010) considering the oligotrophic nature of the glacial photic environment which could be expected to favor metabolically flexible competition specialists (Polz et al., 2006; Hell et al., 2013).

### Implications of Low Viral Decay Rates: Promoting Contact with Host Cells

Supraglacial viruses face the twin challenges of low host cell densities and resource limitation in oligotrophic conditions (Säwström et al., 2007, 2008), impinging upon host–virus contact rates and the proliferation of viral progeny. The maintenance of very high VBR maximizes host–virus contact rates (Anesio et al., 2007) and may be supported by high rates of viral infection typically found in supraglacial environments (Säwström et al., 2007). The physical stability of the supraglacial viral population is very high, with a decay rate of 0.0007 h^-1^ (**Figure 1**); thus, the predicted half-life of viruses in meltwater on the surface of ML would be 39 days. The bare ice season on ML at the altitudes of the sampling and incubation sites in 1998 and 2000 measured 60–68 days and 50–59 days, respectively (Hodson et al., 2007). Therefore, viruses produced during the early stages of the melt season could remain stable for much of a typical growth season on ML, percolating within the interstitial spaces (Mader et al., 2006) of the fractured ice of the weathering crust (Müller and Keeler, 1969), thus promoting the likelihood of contact with bacterial hosts present in the weathering crusts (Irvine-Fynn et al., 2012). It should be noted that the prolonged stability of supraglacial viruses has implications for downstream environments. Nevertheless, if viruses liberated from melting glacial ice enter hydrological flow paths with rapid transit times, they can be transported to the glacier fore field and coastal environments. Melt streams on ML exhibit flow rates in the order of 0.5–0.6 m s^-1^ (Irvine-Fynn et al., 2005), delivering viruses from the sampling site to the glacier margin in 60–70 min. The stability of the supraglacial viruses incubated in proglacial lake conditions (mean *k* = 0.001 ± 0.006 h^-1^) implies the viral community is resilient to temperature changes associated with such transfers, and the experimental infection of downstream bacteria by supraglacial viruses (Anesio et al., 2007; Bellas et al., 2013) has been shown to occur.

### Implications of Low Viral Decay Rates: Viral Control of Bacterial Production

While the prolonged half-life of VLPs presents a mechanism to maintain high VBR, it also necessitates a trade-off between survival and reproductive rate in that additional resources are required in the construction of robust viral capsids and densely packaged genomes which restrain the BS of phage (De Paepe and Taddei, 2006). Considering that high VBRs are not maintained in response to resource enrichment (**Figure 3**), it is therefore likely that life-history traits favoring phage survival over profligate reproduction are responsible for the very low BS (Säwström et al., 2007) found on glacier surfaces. In turn, this trade-off demands considerable viral control of bacterial secondary production to achieve a stable state. From the model presented herein, it appears up to 35.5% of bacterial production in the glacial photic zone of ML is controlled by viruses, resulting in a potent viral shunt in the pool of dissolved organic matter. This value exceeds the top-end estimates of viral control in other polar aquatic ecosystems by up to 5.5% units (Pearce and Wilson, 2003) but is broadly in line with more recent global estimates of viral controls within fluvial systems derived from meta-analysis (Peduzzi, 2015) and is reasonable compared with the earliest estimates of viral control which outstripped cognate rates of bacterial production several folds (Bratbak et al., 1992). The shunt of approximately one-third of the bacterial production via viral lysis to dissolved organic matter is likely to significantly attenuate the flow of carbon to higher trophic levels and modulate bacterial growth efficiencies on glacial surfaces in a similar manner to viral shunts in cryoconite granules on glacier surfaces (Bellas et al., 2013) as well as other aquatic environments (Miki et al., 2008; Motegi et al., 2009). Thus, viral controls on the glacial photic zone are likely to be important influences on the quality and quantity of dissolved organic carbon exported to other (e.g. sensitive coastal water; Hood et al., 2009) ecosystems.

### Nutrient Amendment Alleviates Viral Control of Supraglacial Bacterial Communities

In the broadest terms, nutrient enrichment radically altered VBR and BCP, beyond that observed within the non-amended control incubated in parallel. While the experiment was designed to present scenarios within the range of C, N, and P concentrations measured in Svalbard supraglacial meltwaters at the time it was conducted in 2006 (Säwström et al., 2002), Enrichments IV–VI presented scenarios beyond this range of measured concentrations. Subsequently, others (Zarsky et al., 2013) have reported C and N concentrations in the range of Enrichments IV–VI from Svalbard supraglacial meltwater, illustrating the extended dynamic range of solute resource availability on High Arctic glaciers, which are influenced by deposition of atmospheric nitrogen from lower latitudes (Hodson et al., 2010; Hell et al., 2013). Therefore, while the upper-end enrichment scenarios may be less representative of typical resource availabilities on ML, they are not entirely unrealistic.

Although the results above regarding the viral control of the *in situ* bacterial community are consistent with earlier work (Anesio et al., 2007), addition of nutrients appears to release a fraction of the bacterial community of the glacial photic zone from viral pressure during the course of the experiment. While VLP abundance increases over time (**Figure 2**), the increase factor within the bacterial community is far higher and hence, VBRs decline profoundly (**Figure 3**) in all treatments over time. Furthermore, there is evidence of a nutrient-dependent and time-dependent effect in the VBR decline. Coupled with the trends in bacterial and VLP abundance (**Figure 2**), the strong nutrient-dependent and time-dependent increases in BCP (**Figure 4**) in the experiment demonstrate that a fraction of the bacterial community is able to access the additional resources, while the viral community is unable to retain its control of the bacterial community across time and enrichment treatments (**Figure 3**). This is further evidenced by the strong and highly significant negative correlation between VBR and BCP (**Figure 4H**; Spearman rho = -0.81, *p* < 0.001).

### Insights to Supraglacial Bacterial Community Composition

Logistical considerations precluded the parallel incubations of large volume sacrificial microcosms necessary to assess changes in bacterial (or viral) community composition over time. However, pooling of experimental replicates onto the same filter (required to assure sufficient biomass for DNA extraction; Rassner, unpublished data) at the termination of the experiment and subsequent 16S rRNA gene amplicon pyrosequencing presents some key insights into the overall effects of the nutrient enrichment experiment on the bacterial community. Archaeal community sequencing was not investigated since other studies have failed to detect Archaea in Svalbard glacial melt (e.g. Hell et al., 2013).

Chief among these insights is the dominance of the community by a small core of OTUs, with the 12 OTUs present at ≥1% of mean relative abundance across the experiment accounting for at least half of the relative abundance of OTUs (and ≥84% of the relative abundance of OTUs in the Enrichment treatments). These OTUs predominantly possess CNR isolated from the global cryosphere, typically at ≥99% identity, suggesting that at the end of the experiment, all treatments were dominated by taxa authentic to supraglacial environments (**Figure 5**). Indeed, these same taxa also dominate the source bacterial community profiled in the descriptive sample. It is clear that the release of viral control subsequent incubation under a range of nutrient enrichment scenarios has not incurred a shift in the identity of the dominant OTUs.

Of the 12 OTUs present at ≥1% relative abundance, the highest, 3rd, 4th, and 10th highest relative abundance OTUs all possess CNR within the betaproteobacterial genus *Janthinobacterium* at 95–99% identity. This is reflected by their GreenGenes taxonomic assignments to the genus or its parent family *Oxalobacteraceae* at a confidence of 0.80. The prominence of the most dominant OTU (*Janthinobacterium-*5789) which exhibits 99% identity to a *Janthinobacterium* sp. isolated from an Italian glacier (*Janthinobacterium* sp. UC7209 KJ729009.1; Cappa et al., 2014) is striking, ranging from 41 to 76% relative abundance in the incubations and on average representing over half of the bacterial community. It should be emphasized that this is also the dominant OTU in the *in situ* community, at a relative abundance of 14%. Importantly, the same taxon is dominant in the source community, non-amended control, and Enrichments I–V (**Figure 6**) when the sequence data set is reprocessed using 99% identity OTUs, with the representative sequence of 99*Janth*_24369 showing 100% identity to the 97% identity OTU *Janthinobacterium*-5769 and showing 99% identity to the *Janthinobacterium* sp. UC7209 glacial isolate above. A closely related OTU (**Figure 6B**, 99*Janth*_2653) exhibits an increased relative abundance in Enrichment V and Enrichment VI.

*Janthinobacterium* sequences are found in environmental gene surveys of supraglacial habitats including supraglacial streams (Segawa et al., 2005; Smith et al., 2013; Cappa et al., 2014). The dominance of *Betaproteobacteria* (to which *Janthinobacterium* is affiliated) is consistent with other studies of 16S ribosomal RNA genes and metagenomes from supraglacial habitats (Edwards et al., 2013a; Hell et al., 2013) but not with cryoconite from the same glacier, which was previously found to be dominated by *Alphaproteobacteria* (Edwards et al., 2011, 2013c, 2014b).

### *Janthinobacterium*: It Pays to Be a Winner?

Given the oligotrophic nature of glacier surfaces, the predominance of one taxon, able to outcompete other taxa to access limited resources, might be expected (Winter et al., 2010). However, the high VBR and viral control of bacterial production would imply that a numerically dominant successful competitor would likely suffer the winner’s fate in falling victim to its associated viruses. The continued dominance of *Janthinobacterium*-5789, the experiment, and source community are therefore notable. Here we identify factors which may account for *Janthinobacterium*’s dominance across a range of viral pressure and nutrient availability scenarios.

*Janthinobacterium* itself is noted for its production of defensive antimicrobial compounds, most notably violacein, which protects its symbiotic partners from fungal disease (Brucker et al., 2008; Harris et al., 2009) and is associated with the survival of *Janthinobacterium* in hostile conditions (Pantanella et al., 2007). However, analyses of four glacial *Janthinobacterium* genomes (Kim et al., 2012; Smith et al., 2013) reveal the presence of the violacein biosynthesis cluster is highly variable; therefore, the putative role of defensive antimicrobials in suppressing competing taxa cannot be inferred here. Similarly, the presence of CRISPR mechanisms within the *Janthinobacterium* genomes is variable. Finally, grazer–bacterium–virus interactions are purposefully minimized within the experimental design presented, where the source meltwater was pre-filtered to exclude protozoa.

In the Arctic Ocean, blooms of *Janthinobacterium* and the associated secretion of a mucilaginous surface layer have been described to promote biofilm formation (Alonso-Sáez et al., 2014). We note that the production of extracellular vesicles by *Janthinobacterium* (Hornung et al., 2013), also observed qualitatively here (**Figure 7**; **Supplementary Figure S2**), offers a potential viral evasion mechanism similar to marine cyanobacterial “decoy” vesicles which distort host–virus contact rate (Biller et al., 2014). Although the production of vesicles may appear an expensive defense mechanism, the costs of their production may be offset by their multifarious utilities, for example, combining viral evasion with antimicrobial resistance, biofilm production, and predation (Mashburn-Warren et al., 2008; Manning and Kuehn, 2011; Whitworth, 2011). Even if the extrusion of surface mucilage associated with biofilming or vesicles are not the forms of anti-viral defense principally responsible for the dominance of *Janthinobacterium*, the predominance in glacial meltwater of taxa exuding these forms of colloidal organic matter has implications for the quantity and provenance of organic carbon exported in glacial meltwater (Hood et al., 2015). Future work could investigate the relative contributions of dissolved organic carbon (DOC) liberated from lytic infections and abundant host secretions associated with defense against viral and abiotic stresses. Similarly, a variety of unusual infective strategies aimed at increasing intracellular persistence have been associated with cryoconite viruses, as well as phage putatively infecting members of *Cyanobacteria*, *Actinobacteria*, and *Alphaproteobacteria* typical of cryoconite microbiota (Bellas et al., 2015). However, the metavirome did not identify phage sequences associated with *Betaproteobacteria* more commonly associated with snow (Hell et al., 2013) or ice melt. Future work could target meltwater metaviromes and isolation of *Janthinobacterium*-phage pairs to characterize specific interactions between phage and *Janthinobacterium.*

## Summary

The experimental outcomes of this study provide insights into viral and bacterial strategies within the glacial photic zone. Firstly, viruses exert a strong control on bacterial production, abundance, and diversity within the supraglacial environment. This has implications for the retention and accumulation of bacterial biomass and hence biological darkening at the ice surface (Irvine-Fynn et al., 2012; Irvine-Fynn and Edwards, 2014). It appears that the viral community maintains this pressure by means of prolonged longevity of viral particles. However, the promotion of phage survival, as evidenced by this study, over reproduction (Säwström et al., 2007) presents a trade-off for viruses as producing robust viral capsids and densely packaged genomes constrains the BS of phage (De Paepe and Taddei, 2006). This allows bacteria to evade viral pressure by accessing resources in the form of dissolved nutrients across a spectrum of nutrient amendment scenarios; the mechanisms by which *Janthinobacterium* species retain their dominance of meltwater bacterial communities require further investigation. While our results are constrained to one site, this interplay of bacteria, nutrients, and viruses could influence bacterial biomass accumulation in the glacial photic zone (Irvine-Fynn et al., 2012) and export of organic matter (Hood et al., 2015) to sensitive downstream ecosystems.

## Author Contributions

SR, AA, and AE designed study. SR, AA, and AE conducted fieldwork. SR conducted lab analyses. SG, KH, and JG conducted high-throughput sequencing. SR, JG, DW, and AE performed data analysis. AE and SR wrote manuscript. All authors contributed to manuscript writing and revision.

## Conflict of Interest Statement

The authors declare that the research was conducted in the absence of any commercial or financial relationships that could be construed as a potential conflict of interest.
