# Supplementary material for: Can the Bacterial Community of a High Arctic Glacier Surface Escape Viral Control?
Source: Front Microbiol. 2016 Jun 21;7:956. doi: 10.3389/fmicb.2016.00956 (PMC4914498; doi:10.3389/fmicb.2016.00956)
Supplement: Supplementary file 1 [file Data_Sheet_1.DOCX]

Supplementary Material

Can the bacterial community of a High Arctic glacier surface escape viral control?

**Sara M. E. RASSNER^1, 2^, Alexandre M. ANESIO^3^, Susan E. GIRDWOOD^1^, Katherina HELL4, Jarishma K. GOKUL^1^, David WHITWORTH^1^, & Arwyn EDWARDS^1*^,**

**1 Institute of Biological, Rural and Environmental Sciences, Cledwyn Building, Aberystwyth University, Aberystwyth, SY23 3FG, UK.**

**2 Department of Geography and Earth Sciences, Llandinam Building, Aberystwyth University, Aberystwyth, SY23 3DB, UK.**

**3Bristol Glaciology Centre, School of Geographical Sciences, University of Bristol, University Road, Bristol, BS8 1SS, UK**

**4 Institute of Ecology, University of Innsbruck, Technikerstrasse 25, 6020 Innsbruck, Austria.**

*** Correspondence:**  Arwyn Edwards. Institute of Biological, Rural and Environmental Sciences, Cledwyn Building, Aberystwyth University, Aberystwyth, SY23 3FG, UK.

aye@aber.ac.uk

# Supplementary Data

Viral Decay Modelling

Bacterial growth rates (*µ_b_* in units of h^-1^) were calculated on the assumptions of 11 fg C cell^-1^ (Norland et al 1987) multiplied by bacterial abundance (BA) in cells per millilitre to provide bacterial biomass (BB) using the approach of Crump *et al.* (2007):

$$\mu_{b}=\ln\left( \frac{BB+BCP}{BB} \right)$$

**Eq.1.**

The decay data were subjected to natural logarithm transformation to achieve homogeneity of variance and covariance (sphericity) and analyzed for statistically significant differences (p<0.05) between community sources, locations of incubation and treatments (between-subject factors) and over time (within-subject factor) using repeated measures analysis of variance (rm-ANOVA; SPSS for Windows versions 15.0 & 17.0). To investigate the dynamics of viruses in the habitats investigated, the model of Fischer *et al.* (2004) for the steady –state production and decay of viral particles was applied:

$$\frac{d\mathrm{VA}}{dt}=\left( BS\times f_{v}\times BA \right)-\left( k\times VA \right)=0$$

**Eq.2.**

Where changes in viral abundance (*VA*) over time (*t*) are regulated by the product of *VA* and the viral decay rate (*k,* in units of h^-1^) subtracted from the production of fresh virus, given by the product of the burst size (*BS*), bacterial abundance (*BA*) and the rate of infection of cells leading to production of new virus (*f_v_*_,_ viral proliferation) was applied. Since cells were excluded from the experimental units by filtration through 0.2 µm filters and changes in bacterial abundance monitored, it is assumed viral production in microcosms not showing growth of cells is negligible; the terms for viral production are disregarded, ending the steady state and resulting in both the gross and net decay of viral particles:

$$\frac{d\mathrm{VA}}{dt}=-k\times\mathrm{VA}$$

**Eq. 3.**

Which can be represented as an exponential decay function, taking the form:

$$\mathrm{VA}_{t}={VA}_{0}\times e^{-kt}$$

**Eq. 4.**

Where the viral abundance (VLP mL^-1^) at time *t* is given by *VA_t_* and initially by *VA_0_*.

Within this experiment, values of *k* were tested for statistically significant differences (*p*<0.05) between community sources, locations of incubation and treatments (between-subject factors) using Kruskal-Wallis tests (Minitab Release 14.2). The calculation of *k* is limited in its utility for comparison with other published studies of viral decay experiments since different methods of calculation result in considerable variation in values of *k* even when utilizing data from within the same experiment if different models are applied (Fischer et al 2004). However we do present an estimation of the rate of viral proliferation using the method of Fischer *et al.* from calculated values of *k* (equalized to units of h^-1^ on the assumption of minimal diurnal variation in rates of decay)*,* measurements of *VA* and *BA* in descriptive samples of water from this study and a mean *BS=3* on the basis of values reported from supraglacial meltwater on Midtre Lovénbreen sampled in July 2005 by Säwström *et al.* (2007):

$$f_{v}=\frac{(k\times VA)}{(BS\times BA)}$$

**Eq. 5.**

This enables the determination of the percentage of bacterial secondary production controlled by viruses (*VC*; (Fischer et al 2004)) as follows using the measurements of bacterial secondary production made in this study to calculate bacterial growth rate and the value for viral proliferation derived above:

$$VC=\frac{f_{v}}{\mu_{b}}\times100$$

**Eq. 6.**

Finally, a crude estimate of the carbon released by a generation of viruses is given as *C_loop_* in units of fg C mL^-1^ where *C_c_* is the cellular carbon content, which is given in the range of 11 fg C cell^-1^ on the basis of values utilized by other workers (Anesio et al 2010, Priscu and Christner 2004) and *C_v_* is the carbon content of an aquatic VLP at 0.2 fg C VLP^-1^ as per Suttle (2005).

$$C_{loop}={{BA \times f}_{v}(C}_{c}-{[C}_{v}\times BS])$$

**Eq. 7.**

Scanning Electron Microscopy (SEM)

Samples of meltwater subjected to SEM to optimize sample processing for a different experiment (S.M. Rassner, [2009] unpublished Ph.D. thesis, Aberystwyth University) provided an opportunity to interrogate the cellular morphology of bacteria in the nutrient enrichment experiment. All SEM analyses were conducted at the Aberystwyth University IBERS Bioimaging Laboratory with the support of Dr. Iolo ap Gwynn and Dr. Stephen C. Wade.

Autoclaved glass mirror discs (Panduro Hobby AB, Malmö, Sweden, Catalogue number 042791) were soaked overnight in a strong VIRKON solution made up with Milli–Q water and rinsed in Milli–Q water before air-drying and rinsing with 80% ethanol. To test the effectiveness of the cleaning procedure, six discs were placed in a closed Petri dish, covered with a liquid culture of bioluminescent bacteria (*Vibrio harveyi,* courtesy of Dr. H. P. Moore, IBERS) and left to stand in the dark at room temperature for 6 h. Three discs were examined for bioluminescence straight away and three discs were examined after having been soaked in a strong VIRKON solution overnight. There were no visible cells on the cleaned discs and there was no evidence of bioluminescence. Thus, the cleaning procedure was deemed sufficient. The discs were coated with gold (thickness: 40–50 nm) using a sputter coater (Polaron, Quorum Technologies) with an argon gas environment. The resulting Au coat was sufficiently thick to prevent charging of the specimen.

The disc was attached to a conical holder using a small amount of white tack (W H Smith Ltd.) and the holder was placed in a 50 ml Falcon tube. The holder had been made out of epoxy resin using the conical bottom part of a 50 ml Falcon tube as a mould and held the disc securely and parallel to the opening of the tube, thus ensuring a uniform distribution of cells over the disc when centrifuged in a swing bucket centrifuge.

The sample was thawed at +4°C and up to 45 ml of sample was transferred to the prepared Falcon tube. The sample volume was adjusted so that the disc was covered in a monolayer of well-dispersed cells. Preliminary tests indicated an upper limit of ca. 2×10^7^ cells on the discs for clear separation of cells during the SEM analysis. Since the area of the disc covered 27% of the area of the holder, the volume of sample added to the tube should contain no more than ca. 8×10^7^ cells.

The cells in the sample were spun down onto the Au-coated disc for 1 h at 5850×*g* min^-1^ at +10 C, using a swing bucket centrifuge (Multifuge 3 S–R, Heraeus Instruments). After centrifugation the liquid was carefully decanted and the disc removed from the holder. The side of the disc was touched against a piece of blue roll and the disc was left to air dry in a closed Petri dish in the dark.

The dry disc, carrying the sample, was glued onto an aluminium specimen stub (­12.5 mm; Agar Scientific Ltd.) using quick-drying silver paint (Agar Scientific Ltd.). The side of the disc was painted with Ag paint, thereby forming a conductive bridge between the aluminium stub and the Au-coating. The silver paint was allowed to dry for 1–2 d, before staining with 2% (w/v) uranyl acetate dissolved in methanol.

The sample was analysed by SEM (Hitachi s–4700 II FE–SEM), using the ultra-high resolution operation mode, with a specimen bias voltage of -40 V, an accelerating voltage of 0.8 kV and an emission current of 30 mA. The working distance was 1.4 mm (± 3 mm).

Images were captured by the Hitachi FE–SEM PC software using the slow image capture option (capture resolution: 2560×1920) and transferred to Quartz PCI (Quartz Imaging Corporation). Image contrast and brightness were adjusted automatically, or manually using the histogram function. Micrographs were saved in JPEG format and viewed on a computer monitor in Microsoft Office Picture Manager or Adobe Photoshop CS3.

Anesio AM, Sattler B, Foreman C, Telling J, Hodson A, Tranter M *et al* (2010). Carbon fluxes through bacterial communities on glacier surfaces. *Annals of Glaciology* **51:** 32-40.

Caporaso JG, Kuczynski J, Stombaugh J, Bittinger K, Bushman FD, Costello EK *et al* (2010). QIIME allows analysis of high-throughput community sequencing data. *Nature Methods* **7:** 335-336.

Crump BC, Peranteau C, Beckingham B, Cornwell JC (2007). Respiratory Succession and Community Succession of Bacterioplankton in Seasonally Anoxic Estuarine Waters. *Applied and Environmental Microbiology* **73:** 6802-6810.

Fischer UR, Weisz W, Wieltschnig C, Kirschner AKT, Velimirov B (2004). Benthic and Pelagic Viral Decay Experiments: a Model-Based Analysis and Its Applicability. *Applied and Environmental Microbiology* **70:** 6706-6713.

Norland S, Heldal M, Tumyr O (1987). On the relation between the dry-matter and volume of bacteria. *Microbial Ecology* **13:** 95-101.

Priscu JC, Christner BC (2004). Earth’s icy biosphere. In: Bull AT (ed). *Microbial diversity and bioprospecting*. American Society for Microbiology: Washington D.C., USA. pp 130-145.

Suttle CA (2005). Viruses in the sea. *Nature* **437:** 356-361.

Säwström C, Granéli W, Laybourn-Parry J, Anesio AM (2007). High viral infection rates in Antarctic and Arctic bacterioplankton. *Environmental Microbiology* **9:** 250-255.

Supplementary Figures and Tables

## Supplementary Figures


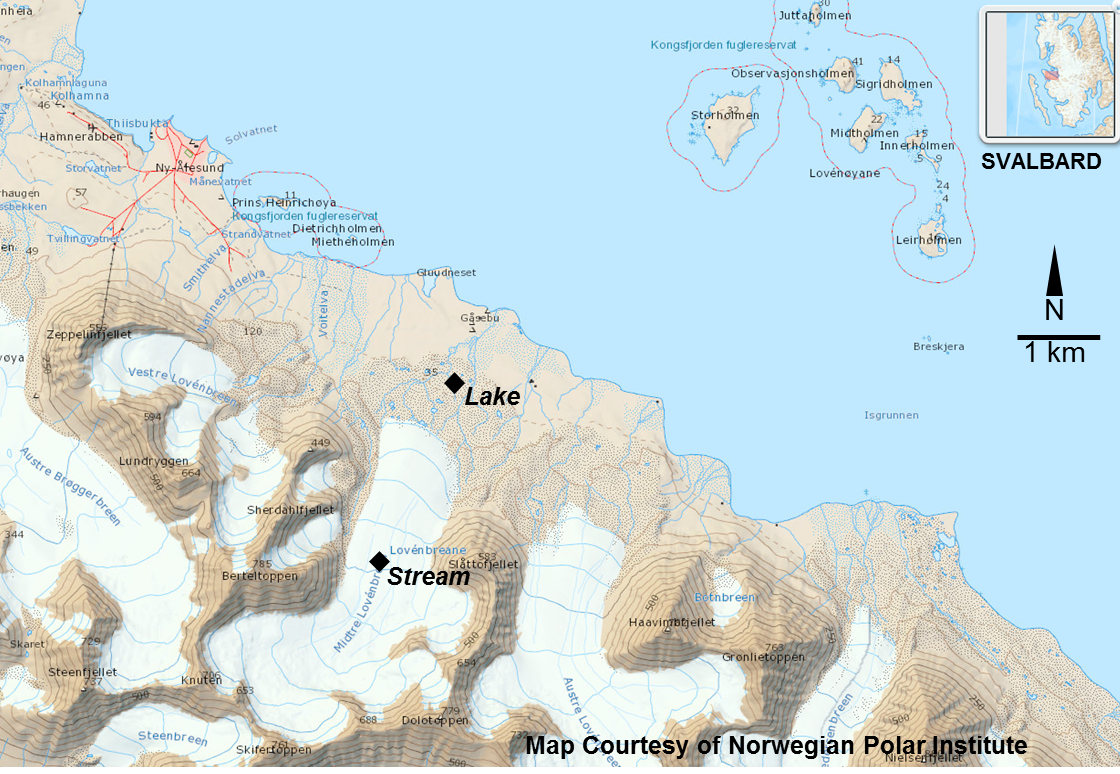


**Supplementary Figure 1**: Map of study area illustrating sampling points associated with ML

**
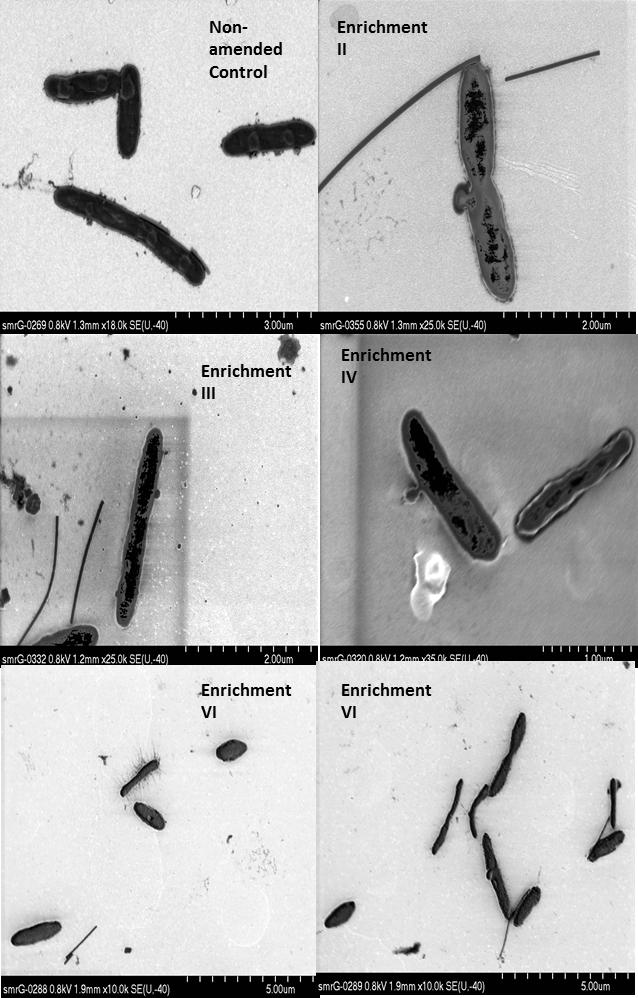
**

**Supplementary Figure 2**: Selection of SEM images of bacteria exhibiting features consistent with extracellular vesicles.
